# Myocardium at risk and myocardial salvage after acute infarction in humans; quantification by magnetic resonance imaging

**DOI:** 10.1186/1532-429X-11-S1-O29

**Published:** 2009-01-28

**Authors:** Marcus Carlsson, Joey Ubachs, Einar Heiberg, Erik Hedstrom, Stefan Jovinge, Hakan Arheden

**Affiliations:** 1 Clinical Physiology, Lund University Hospital, Lund, Sweden; 2 Cardiology, Lund University Hospital, Lund, Sweden

**Keywords:** Left Ventricle, Percutaneous Coronary Intervention, Infarct Size, Heart Beat, Independent Method

## Introduction

To assess reperfusion therapy, it is necessary to determine how much myocardium is salvaged by measuring the final infarct size in relation to the initial myocardium at risk (MaR) of the left ventricle (LV). T2 weighted MRI has recently been shown to be able to identify MaR but has not been validated against an independent method in humans.

## Purpose

To validate myocardium at risk by T2 weighted MRI (T2-STIR) over time, compared to perfusion SPECT in patients with ST-elevation myocardial infarction (STEMI), and, to assess the amount of salvaged myocardium after 1 week.

## Methods

Sixteen patients with first-time STEMI received ^99m^Tc tetrofosmin prior to primary percutaneous coronary intervention and SPECT was performed within 4 hours to identify MaR. MRI was performed on a 1.5 T Siemens Vision (5 patients) or Philips Intera. T2-STIR was at 1 day, 1 week, 6 weeks and 6 months. Image parameters for T2-STIR were: TE, 43 ms (Siemens), or 100 ms (Philips); TR, 2 heart beats; NEX, 2; image resolution, 1.5 × 1.5 mm; slice thickness, 10 mm (Siemens) or 8 mm with a slice gap of 2 mm (Philips). At 1 week, patients were injected with a gadolinium-based contrast agent for quantification of infarct size on delayed contrast enhanced (DE) MRI. T2-STIR and DE MR images were acquired in the short-axis view, covering the left ventricle from the base to apex.

## Results

**Figure 1:**
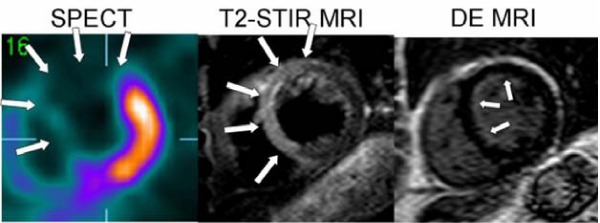
Figure 1

MaR at occlusion on SPECT was 33 ± 10% of the LV. MaR on T2-STIR did not differ from SPECT, at day 1 (29 ± 7%, p = 0.74), or week 1 (31 ± 6%, p = 0.23) but declined at week 6 (10 ± 12%, p = 0.03 vs. 1 week) and month 6 (4 ± 11%, p = 0.02 vs. 1 week). The difference between SPECT and T2-STIR at 1 week was -2.3 ± 5.7%. Both modalities identified MaR in the same perfusion territory and in concordance with coronary angiography. Final infarct size was 8 ± 7% and salvage was 75 ± 19% of MaR. Figure 1.

## Conclusion

This is the first study to validate T2-STIR for quantification of MaR against an independent method (SPECT) in patients with acute ST-elevation myocardial infarction after reperfusion therapy. The results demonstrate that T2-STIR performed up to one week after reperfusion can accurately determine myocardium at risk as it was before opening of the occluded artery. The result of reperfusion therapy can therefore be assessed clinically by calculating myocardial salvage as the difference between myocardium at risk and final infarct size using MR imaging.

